# Matrix Acidizing in Dolomitic Rocks: Wormhole Development and Injection Performance Analysis

**DOI:** 10.1021/acsomega.6c08425

**Published:** 2026-10-14

**Authors:** Aline Maria Fernandes Galdino da Silva, Ernani Dias da Silva Filho, Guilherme Mentges Arruda, Marcos Allyson Felipe Rodrigues, Mateus Palharini Schwalbert, José Antonio Barbosa, Leonardo José do Nascimento Guimarães, Osvaldo de Freitas Neto

**Affiliations:** † Department of Civil and Environmental Engineering, 28123 Federal University of Rio Grande do Norte, 59078-970 Natal, Brazil; ‡ Department of Petroleum Engineering, Federal University of Rio Grande do Norte, 59064-970 Natal, RN, Brazil; § Department of Chemical Engineering, Federal University of Rio Grande do Norte, 59072-970 Natal, Brazil; ∥ Petrobras Research Center (CENPES), Petrobras, Rio de Janeiro 21941-915, Brazil; ⊥ Department of Geology, 28116 Federal University of Pernambuco, 50740-530 Recife, PE, Brazil; # Civil Engineering Post-Graduation Program, Federal University of Pernambuco, Recife 50740-530, PE, Brazil; ∇ Institute of Chemistry, Federal University of Rio Grande do Norte, 59078-970 Natal, Brazil

## Abstract

Matrix acidizing is widely used to improve the productivity of carbonate reservoirs, particularly those with significant dolomitic content, which account for a substantial fraction of global hydrocarbon reserves. In this study, the pore volume to breakthrough (PV_bt_) behavior of a dolomitic rock was investigated during acidizing with 15 wt % HCl containing commercial additives, namely a corrosion inhibitor (0.5% v/v) and an emulsion preventer (0.2% v/v). Eight core flooding experiments were conducted at 60 °C under a confining pressure of 2000 psi, a backpressure of 1200 psi, and an axial stress of 500 psi, with injection flow rates ranging from 0.4 to 4.0 mL/min. The samples exhibited porosities from 13.99% to 14.94% and permeabilities between 0.28 and 2.47 mD. The PV_bt_ curve followed the dissolution trend described by the Buijse and Glasbergen model, with an optimum PV_bt_ of 2.03 at 2.0 mL/min, corresponding to the most efficient stimulation condition and the formation of a dominant wormhole requiring the minimum acid volume for breakthrough. X-ray microcomputed tomography images revealed three main dissolution patterns: face dissolution and/or conical wormholes at low flow rates, dominant wormholes with branched structures under optimal conditions, and highly branched wormhole networks at high flow rates. The results demonstrate the strong influence of dolomite dissolution kinetics on acidizing efficiency and highlight the importance of selecting an appropriate injection rate for the stimulation fluid under investigation. This study provides new insights into reactive transport and wormhole development in dolomitic rocks and supports the optimization of acid stimulation treatments in dolomitic carbonate reservoirs.

## Graphical abstract

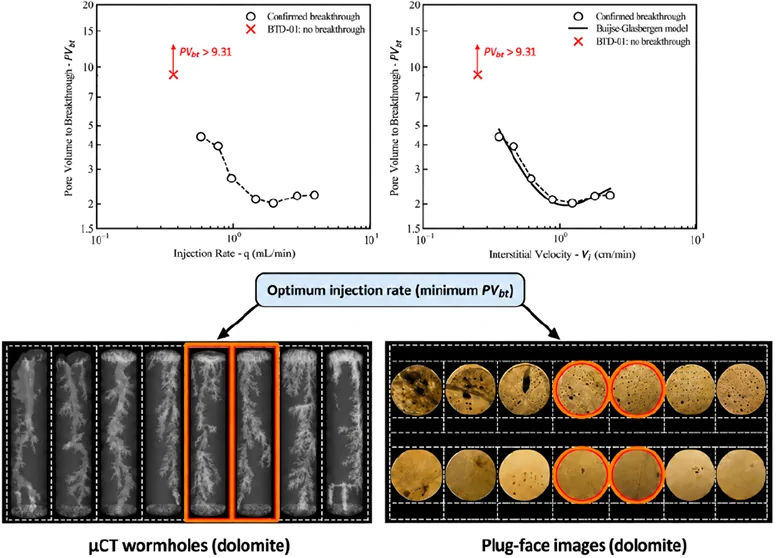

## 1 Introduction

Reservoir rocks play an essential role in the hydrocarbon industry because they can store and transmit oil and natural gas, a behavior directly associated with their favorable porosity and permeability. These formations are mainly composed of sedimentary lithologies, such as sandstones and carbonates, located in the subsurface.^1,2^ Among these lithologies, carbonate rocks host approximately half of the global oil and gas reserves, reinforcing their relevance in global hydrocarbon production systems.^3^

Carbonate rocks are predominantly composed of calcite (CaCO_3_) and dolomite (CaMg­(CO_3_)_2_), minerals that account for most natural carbonate lithologies.^4^ Carbonate reservoirs exhibit mixed mineralogical compositions rather than being composed of a single dominant mineral.^5^ The dolomitization of limestone, a process in which calcium ions are partially replaced by magnesium, results in dolomitic rocks, characterized by more than 50% dolomite content.^6,7^

Physical and chemical properties of these rocks directly influence production efficiency and, consequently, the economic performance of the hydrocarbon recovery operation.^8^ One of the main operational challenges is formation damage, defined as the reduction of permeability around the wellbore caused by mechanisms such as pore throat blockage, fines migration, chemical precipitation, and fluid-rock interactions.^9–11^ To reverse or minimize such damage, stimulation techniques are employed, such as hydraulic fracturing, matrix acidizing, and, especially in carbonates, acid fracturing.

Among these alternatives, matrix acidizing stands out for its simplicity and effectiveness. In carbonate reservoirs, matrix acidizing is commonly used to create pathways and improve fluid flow through the porous medium, thereby increasing reservoir productivity. This technique uses an acid fluid, typically HCl (15% w/w), injected directly into the formation matrix. The reaction promotes the selective dissolution of minerals and creates high-permeability channels in carbonates, known as wormholes, which are responsible for restoring or increasing the formation’s conductivity around the wellbore.^4,12^

Wormhole formation and PV_bt_ behavior are governed by the interplay of several parameters, particularly rock mineralogy, acid formulation, temperature, and flow conditions during acid injection.^13–16^ These variables directly affect the balance between reaction kinetics and mass transfer, thereby controlling dissolution patterns and stimulation efficiency. In this context, PV_bt_ is a key parameter for identifying the optimal acid injection rate at which the smallest acid volume is required to create effective flow channels.

Despite its operational advantages, the use of HCl requires additives such as corrosion inhibitors and emulsion preventer to mitigate damage to the metallic components of the system and prevent the formation of undesired emulsions at the rock-fluid interface.^17,18^ Under reservoir conditions, the products of the reaction between HCl and carbonate minerals are water-soluble, thus preventing secondary precipitations.^17,19^ However, dolomite reacts more slowly with HCl than calcite under equivalent temperature conditions due to its reaction-rate-limited dissolution mechanism, which increases the overall complexity of the acid-rock interaction.^20–22^ The dissolution reaction of dolomite can be represented by eq 1.

1 $$\mathrm{CaMg}{\left({\mathrm{CO}}_{3}\right)}_{2}\left(s\right)+4\mathrm{HCl}\left(\mathrm{aq}\right)\to {\mathrm{CaCl}}_{2}\left(\mathrm{aq}\right)+{\mathrm{MgCl}}_{2}\left(\mathrm{aq}\right)+2{\mathrm{CO}}_{2}\left(g\right)+2H_{2}O$$

Researchers, including Martyushev et al.^5^ and Mustafa et al.,^23^ recently introduced an eco-friendly acid formulation using HCl with additives for the matrix acidizing of carbonate rocks with distinct mineralogical compositions, including dolomites. Their findings indicated that the intrinsic heterogeneity of dolomites presents a more significant challenge for effective stimulation treatments when compared to limestones. Dong et al.^24^ conducted experiments with dolomite at different temperatures using 15 wt % HCl with 0.3 vol % corrosion inhibitor. Their results indicated that dolomite cores require a higher volume of acid to reach the optimum pore volume to breakthrough at lower temperatures (50 °C), with wormhole structures that have larger volumes. Mustafa et al. compared the impact of acid wormhole on the mechanical properties in different mineralogies in matrix acidizing using EDPI acid (20 wt % HCl and 80% other additives).^13^ Across all tested conditions, dolomite required a greater acid volume to breakthrough compared to limestone, characterized by the development of significantly wider dominant wormholes.

Despite advances in the literature, studies investigating high-purity dolomites by integrating PV_bt_ analysis and μCT imaging under representative reservoir conditions remain scarce. Therefore, the understanding of acid-rock interaction and the factors that favor wormhole formation in dolomite rocks is fundamental for improving the efficiency of treatments.

In this context, the present work investigates the influence of the injection flow rate on the reactive transport behavior and dissolution efficiency of dolomites with low permeability. The analysis of the PV_bt_ curve, associated with microtomography images, enables a mechanistic interpretation of wormhole formation patterns under different operational conditions, thereby supporting the optimization of acid stimulation treatments in dolomitic carbonate reservoirs.

## 2 Materials and Methods

The experimental procedures were organized to understand both the intrinsic characteristics of the dolomitic rocks and their response to the acidizing process. Thus, the rock and the acid system were initially characterized and then exposed to core flooding tests conducted under conditions representative of those found in subsurface hydrocarbon reservoirs.

### 2.1 Materials

For the stimulation fluid, analytical-grade hydrochloric acid (HCl, 37% w/w) and two commercial additives (corrosion inhibitor and an emulsion preventer) were used. The corrosion inhibitor serves to protect the metal equipment against deterioration caused by the acid environment, whereas the emulsion preventer prevents the formation of undesired emulsions. Because the core flooding apparatus contains metallic components directly exposed to the stimulation fluid, uninhibited HCl was not circulated through the system to minimize acid-induced corrosion and preserve the integrity of the experimental equipment. Thus, the experimental matrix was designed using the complete stimulation formulation rather than additive-free HCl. Due to confidentiality agreements signed with the supplier, the compositions of these additives cannot be disclosed. However, according to the provided information, the corrosion inhibitor contains aldehydes, primary and secondary alcohols, nonionic surfactants, cationic surfactants, and fatty acid derivatives. The emulsion preventer, in turn, is composed of alcohol and nonionic surfactants. The supplier does not classify this product as a mutual solvent; therefore, no specific mutual-solvent effect was considered in the interpretation of the core flooding experiments. The samples used were carbonate rock plugs of the Bonneterre Dolomite type, acquired from Kocurek Industries, Inc. (Texas, USA), with approximate dimensions of 6″ in length and 1.5″ in diameter, composed predominantly of dolomite.

### 2.2 Stimulation Fluid: Preparation and Characterization

The stimulation fluid was prepared gravimetrically to ensure higher accuracy in additive dosing. In a container, the corrosion inhibitor and the emulsion preventer were added to a volume of distilled water. Subsequently, the solution was supplemented with HCl 37 wt %. Finally, a small amount of distilled water was added to finalize the solution and achieve the desired total volume.

The composition of the acid solution was 15 wt % HCl, containing 0.5 vol % of corrosion inhibitor and 0.2 vol % of emulsion preventer, with the remainder being distilled water. The final stimulation fluid exhibited a measured pH below 0, consistent with the high acidity of the 15 wt % HCl formulation. The density of the acid solution was determined using an Anton Paar DMA 4500 M densimeter, calibrated according to the manufacturer’s protocol at 60 °C.

The fluid viscosity was measured to assess how the additives affect flow behavior and the ability of the solution to penetrate deeper into the rock matrix. The analyses were performed using a Cannon-Fenske capillary viscometer partially submerged in a thermostatic bath at 60 °C. To ensure the accuracy of the results, the time the liquid takes to flow between the two specific marks on the tube was measured in triplicate. The viscosity can be determined using eq 2

2 $$\mu_{s}=\mu_{w}\frac{\rho_{s\cdot }t_{s}}{\rho_{w\cdot }t_{w}}$$

where μ_s_ is the fluid viscosity (cP), ρ_s_ is the fluid density (g/cm^3^), and *t*_s_ is the flow time (s) of the acid solution. The subscripts μ_w_, ρ_w_, and *t*_w_ refer, respectively, to the same parameters for distilled water.

### 2.3 Carbonate Rock Characterization

Representative samples of the Bonneterre Dolomite rock were selected to determine the mineralogical composition. The mineralogical characteristics of dolomite, such as the presence of nonreactive minerals to HCl, can influence the rock’s response to acid treatment, affecting the quantity of CO_2_ released, flow behavior, and modifying the rock’s properties, such as the gain of porosity and, mainly, permeability. The samples were initially crushed and sieved through a 45 μm (325 mesh) screen, producing a fine powder used in the analyses.

The mineralogical composition was determined using X-ray Diffraction (XRD) and X-ray Fluorescence (XRF) techniques. The X-ray diffractogram was obtained using a Bruker D2 Phaser benchtop diffractometer, equipped with a Bruker Lynxeye detector. The analyses were performed at an ambient temperature of 25 °C, employing a Bragg–Brentano geometric angle range (θ–2θ) configuration, with a range of 10° to 80°, steps of 0.02°, and a scan rate of 1°/min. The radiation used was Cu kα (λ = 1.54 Å). The obtained data were interpreted based on the ICSD database and refined using the Rietveld method.

The chemical elements were characterized by X-ray Fluorescence (XRF) using a BRUKER S2 PUMA - SERIES II apparatus, utilizing a Pd tube with a maximum power of 50 W, maximum voltage of 50 kV, maximum current of 1 mA, and a HighSense Silicon Drift Detector (HighSense SDD). To account for compositional variability, three separate samples were macerated, ensuring the representativeness of the results.

Concerning the petrophysical properties of the samples, effective porosity and permeability were determined. These parameters are critical as they directly influence fluid flow dynamics. The detailed experimental procedure is provided in Section 2.5.

### 2.4 Reaction Product Characterization

The products resulting from dolomite reaction in an acid medium were also characterized by XRF. For this, approximately 5 g of powdered dolomitic rock was added to 100 mL of the acid solution containing additives. After 24 h of reaction, solid residues were filtered, washed, and dried in an oven at 60 °C.

### 2.5 Core Flooding Experiments

The experimental methodology consisted of three main stages: preflow, flow, and postflow, as described in Figure 1. In the preflow stage, the selection of the least heterogeneous samples, saturation with brine (KCl 4% (m/v)), and effective porosity measurement were carried out. Next, in the flow stage, the acid fluid was injected at controlled flow rates while monitoring parameters such as pressure, injected volume, and the reaction product. Additionally, the permeability before (*k*_1_) and after (*k*_2_) acid injection was measured. Finally, in the postflow stage, the analysis and interpretation of the PV_bt_ curve data, photographing the faces of the acidified samples, and X-ray microcomputed tomography image generation were performed.

**Figure 1:**
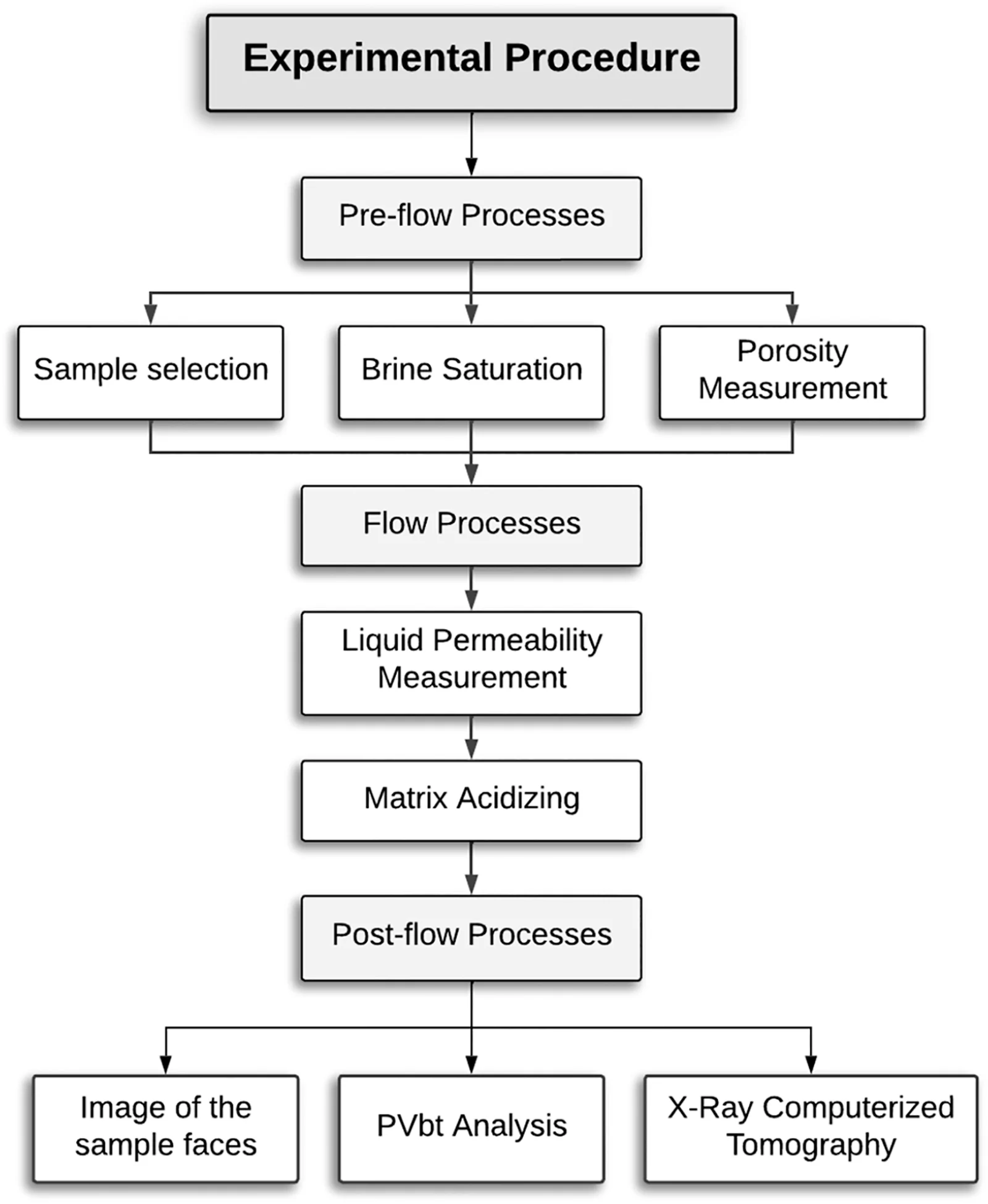
Workflow of experimental procedure.

The samples were saturated before the flow tests using an automatic saturator from Vinci Technologies, with a potassium chloride (KCl) saline solution at a 4% m/v concentration. Knowing the specific mass of the brine and the masses of the samples before and after saturation, it is possible to calculate the pore volume and the effective porosity of the samples, as described by da Silva Filho et al.^25^ The permeability ranges of the samples were obtained at steady state during the brine injection stage of the flow tests.

The Core flooding System (CFS-HM) is shown in Figure 2. This setup is described in detail by Arruda et al.^26^ The sample was wrapped in a heat-shrink sleeve to ensure its integrity and was then transferred to the core holder, where it was subjected to the experiments. The core flooding experiments were conducted at 60 °C using a temperature-controlled oven. Although the stimulation fluid was initially supplied to the injection system at room temperature, it flowed through coiled tubing located inside the oven before reaching the core holder. The residence time in the heated coils allowed the fluid to reach the target experimental temperature before contacting the rock. A thermocouple installed in the acid injection line immediately upstream of the core holder was used to monitor the fluid temperature, confirming that the stimulation fluid reached approximately 60 °C before entering the rock sample. This configuration minimized thermal transients during acid-rock contact and ensured that the acidizing experiments were performed under approximately isothermal conditions at the target temperature. An air-driven liquid pump, manufactured by Magral, was used to apply a confining pressure of 2000 psi on the core holder containing the sample, in addition to a backpressure of 1200 psi.

**Figure 2:**
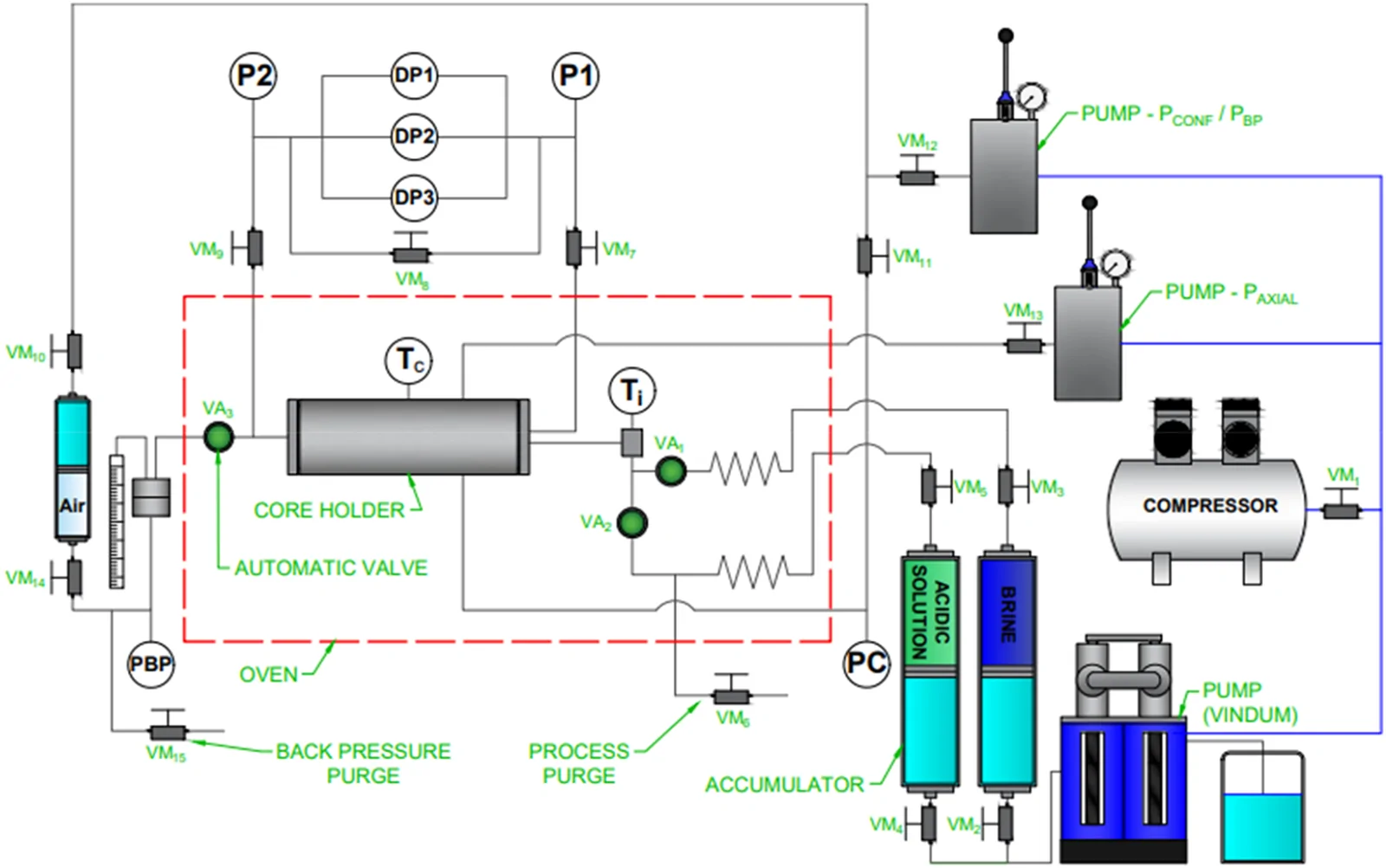
Core flooding System (CFS-HM) setup. Reproduced from Arruda et al.^26^ under the Creative Commons Attribution 4.0 International License.

The brine was injected at a flow rate of 2 mL/min until a steady state was achieved, evidenced by a stable differential pressure across the core. During this stage, the absolute permeability of the rock was determined using Darcy’s Law for linear flow of single-phase, incompressible fluids. Knowing the injection flow rate (q), the brine viscosity (μ) at the experiment temperature, the length and cross-sectional area of the core (*L* and *A*, respectively), and the pressure difference across the plug’s inlet and outlet (Δ*p*), the absolute permeability (*k*) can be calculated, as described by eq 3.^26^

3 $$k=\frac{q\cdot \mu \cdot L}{A\cdot \Delta p}$$

where *k* is the absolute permeability (m^2^), *q* is the volumetric flow rate (m^3^/s), μ is the brine dynamic viscosity (Pa·s), *L* is the core length (m), *A* is the core cross-sectional area (m^2^), and Δ*p* is the differential pressure across the core (Pa). The calculated permeability values were converted to millidarcies (mD) for reporting in Table 3, using 1 mD = 9.8692*·*10^–16^ m^2^.

After the system stabilized, the acidizing stage began, replacing the brine injection with the acid injection. At this point, breakthrough was identified based on two criteria:

i. A change in the pH of the reaction product, indicating the arrival of acid at the sample outlet.
ii. A reduction in the differential pressure between the inlet and outlet of the sample to less than 1% of its initial value.

The effluent pH was used as a qualitative indicator of the arrival of unconsumed acid at the core outlet and was not used directly to calculate PV_bt_ or postacidizing permeability. PV_bt_ was determined from the cumulative acid volume injected at the confirmed breakthrough point, whereas postacidizing permeability was obtained from the subsequent brine-flow pressure response. Thus, breakthrough identification combined chemical and hydraulic criteria, while the absolute pH value was not treated as an independent measure of stimulation efficiency.

After the breakthrough was confirmed, or when an experiment had to be terminated for safety reasons, the acid flow was replaced by brine injection at 10 mL/min to displace and remove residual acid from the sample and the flow lines, thereby minimizing further acid-rock reaction. All faces of the sample were photographed for comparison before and after acidizing. Finally, the samples were conditioned for X-ray microcomputed tomography analyses. Because the experiments were conducted under single-phase brine-saturated conditions, oil–water relative permeability and changes in irreducible water saturation were not evaluated in the present study.

#### 2.5.1 PV_bt_ Curve Analysis

The analysis of the PV_bt_ curve allows for the evaluation of the relationship between the acid injection rate and the volume required for breakthrough to occur in the rock. Based on this, it will be possible to determine the acid consumption and the optimal injection rate, which serves as the primary efficiency indicator for acid stimulation treatment.^14,27^ A total of 8 acidizing tests were conducted across distinct flow rates to construct the PV_bt_ profile.

The pore volume to breakthrough (PV_bt_) is defined as the ratio between the volume of injected fluid (*V*_inj_) and the initial rock pore volume (PV*_i_*), as shown in eq 4.^26^

4 $${\mathrm{PV}}_{\mathrm{bt}}=\frac{V_{inj}}{{\mathrm{PV}}_{i}}$$

This parameter is expressed as a function of flow rate or interstitial velocity (*V_i_*), described by eq 5

5 $$V_{i}=\frac{4q}{{\pi d}_{core}^{2}\cdot \phi }$$

where *V_i_* is the interstitial velocity (cm/min), *q* is the volumetric acid injection rate (cm^3^/min), *d*_core_ is the core diameter (cm), and ϕ is the effective porosity of the core (dimensionless), according to the model proposed by Buijse and Glasbergen.^28^

#### 2.5.2 Microcomputed Tomography

In the X-ray microcomputed tomography (μCT) tests, three-dimensional images are fundamental for analyzing the wormhole formation generated by the acid’s action on the rock. For the rock imaging, a Nikon XT H 225 ST tomograph was used, with a tungsten X-ray source. The projections were generated with an exposure time of 500 ms, totaling an individual projection of acquisition time of 25 min. The electric current was 80 μA, with a 0.25 mm aluminum filter to avoid beam hardening, and the pixel size of all images was 50 μm.

The images were processed using the CTPro 3D software, allowing for the reconstruction of a three-dimensional model of the rock, referred to as the digital rock. Subsequently, the 3D image volumes were analyzed in detail using the VG Studio Max 3.4.4 software, enabling the evaluation of the internal alterations of the rock caused by the acid’s action.

## 3 Results and Discussion

### 3.1 Stimulation Fluid

The viscosity of distilled water at 60 °C was calculated using the viscosity (eq 2) and used as a reference to validate the experimental methodology. The obtained result was 0.466 cP. For the experimental analysis, uncertainty propagation was calculated considering the standard deviations associated with flow time and fluid density measurements.

Table 1 presents the results obtained for the viscosity and density of distilled water, 15 wt % hydrochloric acid, and the stimulation fluid (acid solution containing additives), performed at 60 °C, which was selected as a controlled condition to simulate the operational environment.

**Table 1:** Results for Viscosity and Density for Water, 15 wt % Hydrochloric Acid, and Stimulation Fluid

Parameter	Viscosity (cP)	Density (g/mL)
Distilled Water	0.466	0.9832
HCl 15 wt %	0.698 ± 0.002	1.0550
HCl 15 wt % + Additives	0.718 ± 0.012	1.0555

The density and viscosity results indicate that the addition of the corrosion inhibitor and emulsion preventer produced only small changes in the bulk physicochemical properties of 15 wt % HCl under the investigated conditions. The viscosity increased from 0.698 ± 0.002 cP for 15 wt % HCl to 0.718 ± 0.012 cP for the complete stimulation fluid, while the density changed from 1.0550 to 1.0555 g/mL. Therefore, substantial changes in bulk fluid mobility due solely to viscosity or density are not expected. However, the role of these additives is not limited to their primary operational functions. Although HCl is the reactive component responsible for carbonate dissolution, previous studies from our group have demonstrated that corrosion inhibitors and emulsion preventers can indirectly modify acid-rock interaction, dissolution kinetics, and reactive transport.^25,26^ In particular, Arruda et al.^26^ reported that the presence of these additives reduced PV_bt_ at low injection rates and shifted the optimal interstitial velocity from 0.91 to 0.21 cm/min, corresponding to an approximately 77% reduction. This behavior was associated with a retardation of the effective acid-rock reaction, reducing premature acid consumption near the inlet and allowing deeper penetration of the reactive fluid before breakthrough. From a reactive transport perspective, this retardation can be interpreted in terms of the Damköhler number, which expresses the competition between reaction and transport processes. Possible adsorption and interfacial effects of the corrosion inhibitor and emulsion preventer at the carbonate surface may reduce the effective surface reaction rate and/or mass-transfer rate, thereby decreasing the effective Damköhler number at a given injection velocity. A lower effective Damköhler number reduces premature acid consumption near the inlet and allows deeper penetration of the reactive fluid, which may shift the optimum PV_bt_ condition toward lower interstitial velocities compared with uninhibited HCl. However, because the present study did not measure the individual mass-transfer and surface reaction coefficients, this interpretation should be regarded as a mechanistic explanation rather than a direct quantification of the additive effects.^12^ Therefore, although these additives are not themselves responsible for carbonate dissolution, they may influence the efficiency of acid utilization and wormhole propagation. Nevertheless, because an additive-free HCl core flooding control was not included for Bonneterre Dolomite in the present study, their individual contribution cannot be quantitatively isolated. Thus, the results obtained here should be interpreted as the response of Bonneterre Dolomite to the complete stimulation formulation.

### 3.2 X-ray Diffraction—XRD

Figure 3 presents the results of the XRD analysis performed on the Bonneterre Dolomite rock sample. The diffraction allowed for the identification of the main crystalline planes of the minerals present, providing detailed information about the crystalline structure and structural characteristics of the sample. The data refinement was carried out using the Maud program,^29^ via the Rietveld method,^30,31^ which enabled the precise quantification of the mineral phases present. The analysis of the 2θ angular positions, combined with Rietveld refinement, revealed that dolomite is the predominant mineral phase, with a quantified concentration of 98.5%. This predominance is corroborated by the characteristic peaks of the CaMg­(CO_3_)_2_ crystallographic planes, located at 2θ = 22.04° (101), 30.96° (104), 33.54° (006), 35.31° (015), 37.36° (110) and 43.78° (021).^32,33^ Additionally, a secondary mineral phase corresponding to calcium carbonate (1.5%) was identified, located at 2θ = 29.46°.^34,35^

**Figure 3:**
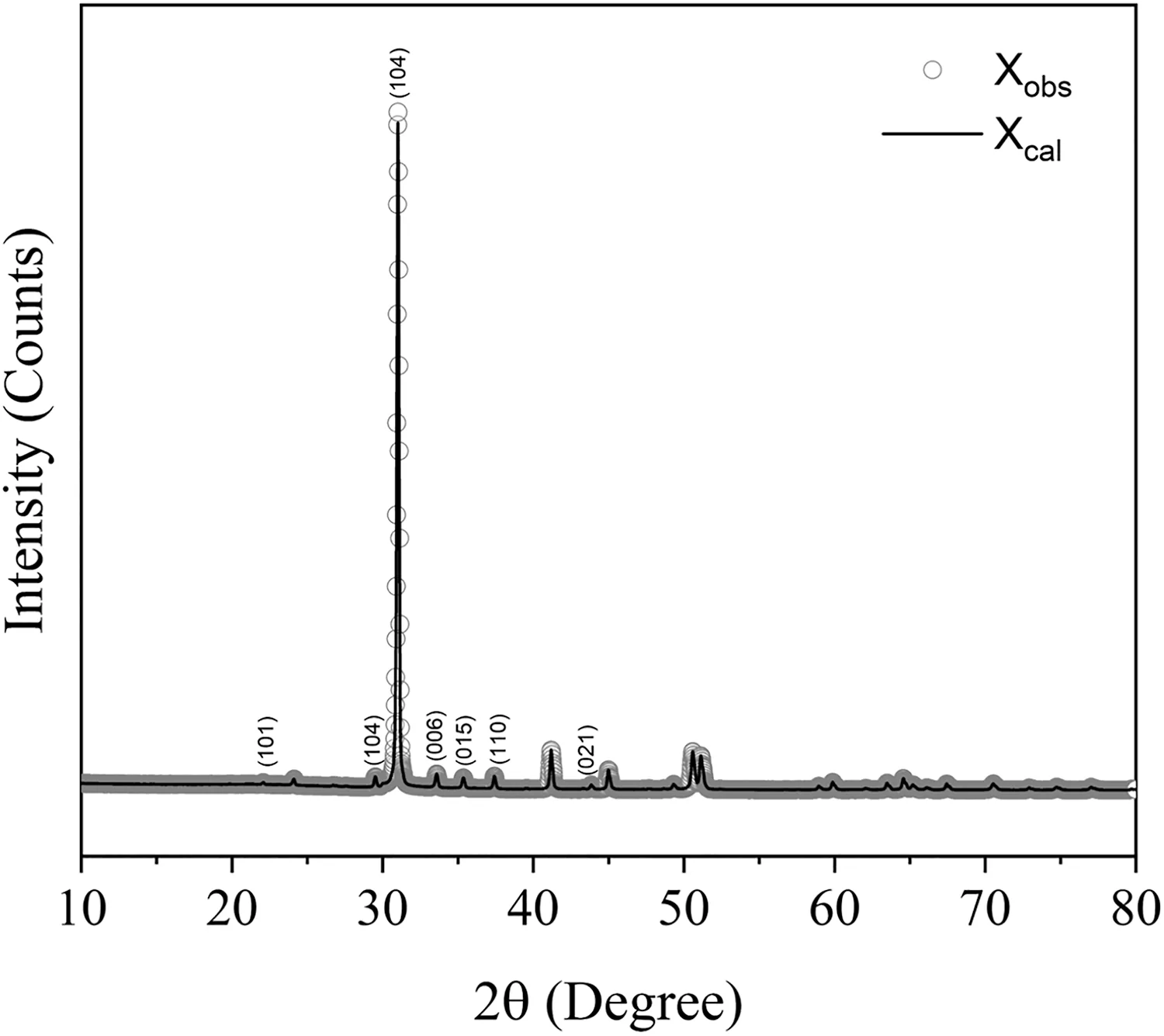
Rietveld refinement profile of XRD data from Bonneterre Dolomite carbonate rock. The circles (gray) correspond to experimental data, and the solid line (black) is the Rietveld refinement.

### 3.3 X-ray Fluorescence—XRF

Table 2 presents the chemical composition of the rock and the reaction product obtained through the XRF technique, with the results expressed in the form of oxides. The quantities of the components are indicated in percentage relative to the total mass. Elements with a concentration lower than 0.1% were omitted to improve data interpretation.

**Table 2:** Chemical Composition (Expressed in Mass %) for the Bonneterre Dolomite Rock

	Composition (% w/w)	
Element	*Bonneterre Dolomite*	Reaction Product
CaO	62.76 ± 1.70	0.16
MgO	28.69 ± 0.52	2.12
Fe_2_O_3_	3.80 ± 0.27	18.45
SiO_2_	1.79 ± 0.41	51.98
Na_2_O	0.90 ± 1.56	-
Al_2_O_3_	0.90 ± 0.14	17.42
MnO	0.59 ± 0.04	0.01
Others	0.57 ± 0.10	9.85
Total	100	100

The Bonneterre Dolomite sample showed significant concentrations of CaO and MgO, indicating the predominance of a calcium–magnesium carbonate phase characteristic of dolomite. These XRF results corroborate the XRD analyses, confirming dolomite as the predominant mineral (98.5%).

Analyzing the results of the reaction product, a solid material resulting from the reaction between the dolomitic rock powder and the acid solution, substantial reductions in CaO and MgO levels confirm the efficient dissolution of the dolomitic matrix (CaMg­(CO_3_)_2_) by the acid solution. In contrast, the relative enrichment of SiO_2_ (51.98%), Al_2_O_3_ (17.42%), and Fe_2_O_3_ (18.45%) suggests that the noncarbonate fraction of the rock remains largely nonreactive to HCl. These results are important for interpreting the core flooding experiments and will be discussed more in Section 3.4.1.

### 3.4 Core Flooding Analysis

#### 3.4.1 PV_bt_ Curve Analysis

A broad range of injection rates was investigated to elucidate the behavior of the PV_bt_ curve. Eight acidizing experiments were performed on Bonneterre Dolomite (BTD) rock samples to determine the PV_bt_ curve using 15 wt % HCl containing additives. It should be noted that an additive-free 15 wt % HCl control was not included in the present core flooding experimental matrix. Therefore, the PV_bt_ and wormhole morphology results discussed herein represent the combined response of Bonneterre Dolomite to the complete stimulation formulation, and the individual contribution of the corrosion inhibitor and emulsion preventer cannot be quantitatively isolated from the intrinsic acid-dolomite reaction. The effective porosity of the samples varied from 13.99% to 14.94%, and the permeability ranged between 0.28 mD and 2.47 mD. The variation in initial permeability should also be considered when interpreting the PV_bt_ results. Although permeability is not explicitly included in the calculation of interstitial velocity, it affects the pressure gradient required to maintain a given injection rate and may influence the accessibility and distribution of reactive flow pathways within the porous medium. Consequently, differences in initial permeability may contribute to variations in acid residence time, local flow redistribution, and wormhole initiation. Nevertheless, the observed stimulation response cannot be attributed solely to permeability, because reactive transport is also controlled by flow conditions, acid-rock reaction kinetics, and intrinsic mineralogical heterogeneity.

Table 3 summarizes the main input parameters and the results for the initial permeability (*k*_1_), the apparent postacidizing permeability (*k*_2_), and the PV_bt_ value.

**Table 3:** Results of Bonneterre Dolomite Core Flooding Experiments at 60 °C

Core ID	Porosity (%)	Rate (mL/min)	Interstitial Velocity (cm/min)	Initial Permeability *k*_1_ (mD)	Apparent postacidizing permeability *k*_2_ (mD)		PV_bt_
BTD-01	14.41	0.4	0.24	0.28	1.48	>9.31^a^	
BTD-02	14.26	0.6	0.37	0.77	3.18 × 10^3^		4.40
BTD-03	14.87	0.8	0.47	0.70	4.58 × 10^4^		3.93
BTD-04	14.03	1.0	0.63	1.28	5.61		2.70
BTD-05	14.57	1.5	0.90	1.05	1.81 × 10^4^		2.12
BTD-06	13.99	2.0	1.25	1.22	6.45		2.03
BTD-07	14.47	3.0	1.82	1.49	2.48 × 10^4^		2.20
BTD-08	14.94	4.0	2.35	2.47	24.7		2.22

a Breakthrough was not achieved. The experiment was stopped after 9.31 injected pore volumes due to safety concerns; therefore, the actual PV_bt_ exceeds 9.31. This data point was not included in the Buijse–Glasbergen model fitting.

In this study, the optimal injection rate was defined based on acid utilization efficiency, corresponding to the condition at which breakthrough was achieved with the minimum injected acid volume. Accordingly, the minimum PV_bt_, rather than the maximum postacidizing permeability, was adopted as the primary optimization criterion. Postacidizing permeability was considered a complementary parameter for evaluating the increase in flow capacity after wormhole formation.

The optimal PV_bt_ value of 2.03 for BTD was observed at an interstitial velocity of 1.25 cm/min. These results are consistent with the literature on dolomitic rocks. Dong et al. reported optimal PV_bt_ values of 2.13 and 0.95 at 50 and 85 °C, respectively, achieved at interstitial velocities of 1.54 cm/min and 4.42 cm/min. Their results indicate that, within this temperature range, PV_bt_ decreases with increasing temperature. Therefore, the behavior observed for BTD at 60 °C (Table 3) is consistent with the expected trend for dolomite acidizing. Although the interstitial velocity corresponding to the optimal PV_bt_ in the present study was lower than that reported by Dong et al.^24^ for nearby temperature conditions, this difference may be attributed to variations in rock properties, acid-rock interaction, and experimental conditions. Overall, the results confirm that the treatment was effective in promoting efficient channel formation at a relatively low acid pore volume requirement.

Mustafa et al. conducted acidizing experiments with an environmentally friendly solution (20 wt % HCl and 80% other additives) in dolomites and limestones at ambient conditions. They found an optimum PV_bt_ value of 2.52 at an injection rate of 1.41 cm^3^/min for dolomitic rock.^23^ The results show higher acid efficiency in limestones compared to dolomites, given that the reaction of dolomite is significantly slower.^20,36^

Two-scale continuum models provide a useful framework for interpreting this behavior because wormhole development is governed by the competition among convective acid transport, mass transfer, and surface reaction kinetics.^36,37^ Numerical sensitivity studies have shown that increasing acid concentration generally decreases PV_bt_ and may shift the optimum injection velocity toward higher values, reflecting the greater availability of reactive species within the porous medium. In the present study, despite dolomite’s low permeability and slower intrinsic dissolution kinetics, 15 wt % HCl was sufficient to generate a well-defined optimum region and a dominant wormhole at intermediate injection rates. At low injection rates, the relatively large acid requirement and the predominance of face or conical dissolution suggest that acid consumption near the inlet limited deeper penetration. Conversely, at higher flow rates, enhanced convective transport promoted the development of thinner and more highly branched dissolution channels. Therefore, the experimentally observed PV_bt_ window is consistent with the reaction-transport competition predicted by continuum models. However, stronger HCl formulations could modify this balance by reducing the acid volume required for breakthrough and shifting the optimum velocity. Consequently, the optimum reported here should be regarded as specific to the investigated 15 wt % HCl formulation and experimental conditions rather than as a universal optimum for dolomitic rocks.

Figure 4a presents the relationship between PV_bt_ and the injection flow rate of the stimulation fluid, whereas Figure 4b presents the same relationship as a function of interstitial velocity. The curve exhibits the characteristic nonlinear behavior associated with carbonate matrix acidizing.^28^ For BTD-01 at 0.4 mL/min, breakthrough was not achieved even after injecting 9.31 pore volumes, and the experiment was interrupted due to safety concerns. Therefore, this condition is reported as PV_bt_ > 9.31 and represents a lower-bound censored observation rather than an exact breakthrough value. Consequently, BTD-01 was not included in the Buijse–Glasbergen regression and is shown in Figure 4 only to illustrate the inefficient acid utilization observed at the lowest injection rate.

**Figure 4:**
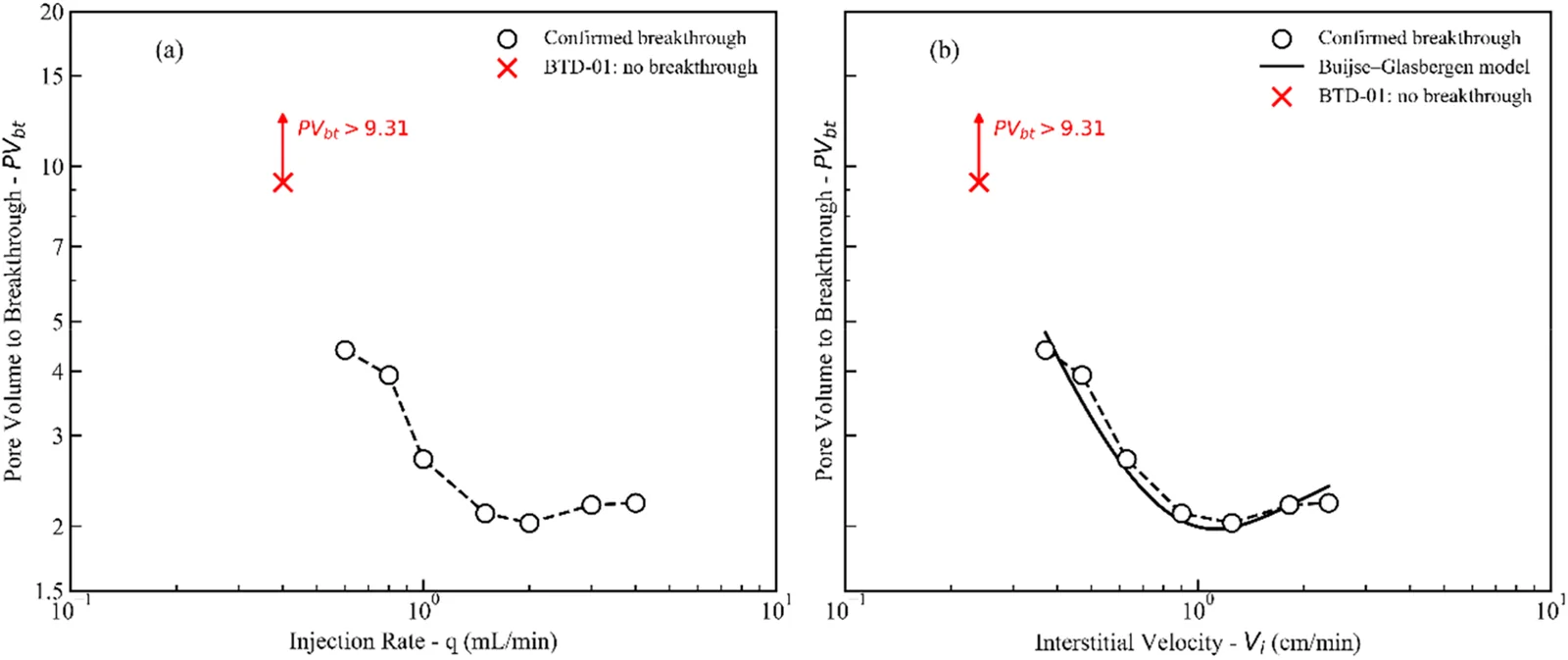
PV_bt_ curves from acidizing tests on Bonneterre Dolomite using 15 wt % HCl with additives as a function of (a) injection rate and (b) interstitial velocity. Symbols denote experimental breakthrough data, and the solid black line in (b) represents the Buijse–Glasbergen model fit obtained using the seven experiments in which breakthrough was confirmed. The × symbol represents BTD-01, for which breakthrough was not achieved after 9.31 injected pore volumes (PV_bt_ > 9.31); this point was excluded from the regression.

The Buijse–Glasbergen model was fitted using the seven experiments in which breakthrough was experimentally confirmed, excluding BTD-01, as it provided only a lower bound for PV_bt_. The revised model provided a good fit to the experimental data (*R* ^2^ = 0.934), indicating that it adequately described the PV_bt_ behavior of the dolomite cores under the investigated conditions. From the fitted form of the model, *W*_eff_ = 0.556 can be interpreted as a parameter associated with the overall scaling of the PV_bt_ response, whereas *W*_B_ = 2.314 is associated with the curvature of the low-velocity branch and, consequently, with the transition toward the optimum acidizing region.^28^ The exponent *n* is an empirical shape parameter associated with the function of the Buijse–Glasbergen model and primarily controls the slope and curvature of the PV_bt_ curve at low interstitial velocities. Higher values of *n* produce a steeper low-velocity branch, whereas lower values provide a smoother transition toward the optimum acidizing region.^26^ In the present analysis, n was fixed at 1 primarily as a modeling constraint to reduce adjustable parameters, particularly because excluding the censored BTD-01 experiment left limited experimental information on the low-interstitial-velocity branch. Therefore, *n* = 1 should not be interpreted as an intrinsic or material-specific value for dolomite. Instead, this assumption provided a smooth representation of the broad experimental transition from inefficient dissolution at low interstitial velocities to the optimum acidizing region without introducing an additional, poorly constrained fitting parameter. Despite excluding the censored BTD-01 condition, the model preserved the characteristic minimum of the PV_bt_ curve in the intermediate-velocity region, consistent with the experimentally observed optimum condition. This interpretation is consistent with the acidizing behavior commonly reported for dolomitic rocks, since dolomite generally reacts more slowly with hydrochloric acid than calcitic rocks and tends to show greater sensitivity to temperature, transport effects, and mineralogical controls during wormhole development.^24,36,38^

Dolomite is a heterogeneous rock, characterized by the presence of insoluble impurities that do not participate in chemical reactions in acid stimulation, due to the presence of elements such as silicates, iron, and aluminum observed in XRF results of reaction products. This characteristic impacts the acid stimulation process in carbonate reservoirs.^39^ For this reason, previous studies have reported greater experimental complexity with this mineralogy compared to limestones.^13^

Monitoring the pressure profile during the core flood experiment is essential for accurately identifying the breakthrough event. From this analysis, the different experimental stages can be clearly identified. The steady state is observed when the system exhibits no variation in differential pressure. The acidizing stage begins when the brine flow is replaced by the stimulation fluid, highlighting the pressure difference. During the initial acidizing phase, the differential pressure increases due to the generation and release of reaction byproducts, such as CO_2_ gas and insoluble or weakly soluble reaction products, which may temporarily obstruct pore throats. Such pressure evolution is consistent with the progressive modification of the pore structure during reactive transport, as predicted by continuum descriptions of carbonate acidizing, in which local dissolution, flow redistribution, and channel growth progressively reduce the core’s hydraulic resistance.

Breakthrough is generally characterized by a pronounced decrease in differential pressure and a substantial increase in flow capacity. In the present experiments, however, the calculated postacidizing permeability values varied by several orders of magnitude. This variability may reflect both experimental uncertainty associated with very low differential-pressure measurements after breakthrough and intrinsic heterogeneity in the dissolution pathways developed within the dolomitic cores. This large dispersion requires cautious interpretation because, after wormhole breakthrough, fluid flow becomes strongly localized within highly conductive dissolution channels. Under these conditions, the assumption of homogeneous porous-medium flow underlying Darcy’s law becomes less representative, and very small differential-pressure values can result in extremely high calculated permeability values. Therefore, the calculated values are interpreted herein as apparent postacidizing permeabilities and as complementary indicators of conductivity enhancement, rather than as the primary criterion for defining acidizing efficiency. In addition, mineralogical heterogeneity and acid-insoluble phases may contribute to the different postacidizing permeability responses observed among the cores. Solid residues were visually observed in the backpressure system (Figure 6). In a separate reaction-product characterization experiment using the same Bonneterre Dolomite and stimulation fluid, XRF analysis showed that the solid fraction remaining after acid exposure was enriched in SiO_2_, Al_2_O_3_, and Fe_2_O_3_. These insoluble phases may remain within pore throats or newly formed conductive pathways, partially limiting the increase in permeability in some samples.^5^ From a field perspective, this behavior may be particularly relevant in heterogeneous dolomitic formations, where acid stimulation may promote carbonate dissolution and wormhole development without necessarily producing a proportional increase in formation conductivity. Local accumulation of acid-insoluble minerals may therefore reduce treatment efficiency and should be considered when selecting acid volumes, injection rates, and post-treatment cleanup strategies. Importantly, this behavior represents a trade-off between acid-utilization efficiency and post-treatment hydraulic performance. A condition that minimizes the acid volume required for breakthrough does not necessarily maximize the effective conductivity created by the treatment. If acid-insoluble mineral phases accumulate within pore throats or newly formed wormholes, the conductive pathways generated by carbonate dissolution may become partially restricted. Therefore, the optimum condition defined by the minimum PV_bt_ should be interpreted as the optimum for acid utilization, while the potential retention or redistribution of insoluble solids represents an important operational risk to consider when translating laboratory results to field-scale acidizing treatments.

The presence of acid-insoluble reaction products and their accumulation in the backpressure system may also introduce additional uncertainty into the postacidizing pressure measurements. However, the amount of solid residue and the degree of obstruction were not quantified individually for each core flooding experiment. Therefore, a direct quantitative relationship between residue accumulation and the individual apparent postacidizing permeability values cannot be established, and the observed *k*_2_ variability should be interpreted as the combined result of pressure-measurement sensitivity after breakthrough, wormhole connectivity, intrinsic rock heterogeneity, and possible local accumulation of acid-insoluble phases. For this reason, breakthrough identification was based on the combined analysis of the differential-pressure response and the change in effluent pH, as described in Section 2.5.

Figure 5 shows the differential-pressure profile of sample BTD-06 at the optimum injection rate of 2.0 mL/min. Although breakthrough was clearly achieved, the apparent postacidizing permeability increased only from 1.22 to 6.45 mD. This comparatively moderate permeability enhancement indicates that breakthrough does not necessarily result in permeability increases of several orders of magnitude. One possible explanation is the persistence and redistribution of acid-insoluble mineral phases during carbonate dissolution. Solid residues were visually observed in the backpressure system. In a separate reaction-product characterization experiment using the same rock and stimulation fluid, XRF analysis showed enrichment of SiO_2_, Al_2_O_3_, and Fe_2_O_3_ in the remaining solid fraction. These residual phases may partially restrict pore throats or conductive pathways, thereby limiting the postacidizing hydraulic response. Therefore, Figure 5 provides a representative example of the complex relationship between breakthrough and postacidizing permeability in heterogeneous dolomitic rocks.

**Figure 5:**
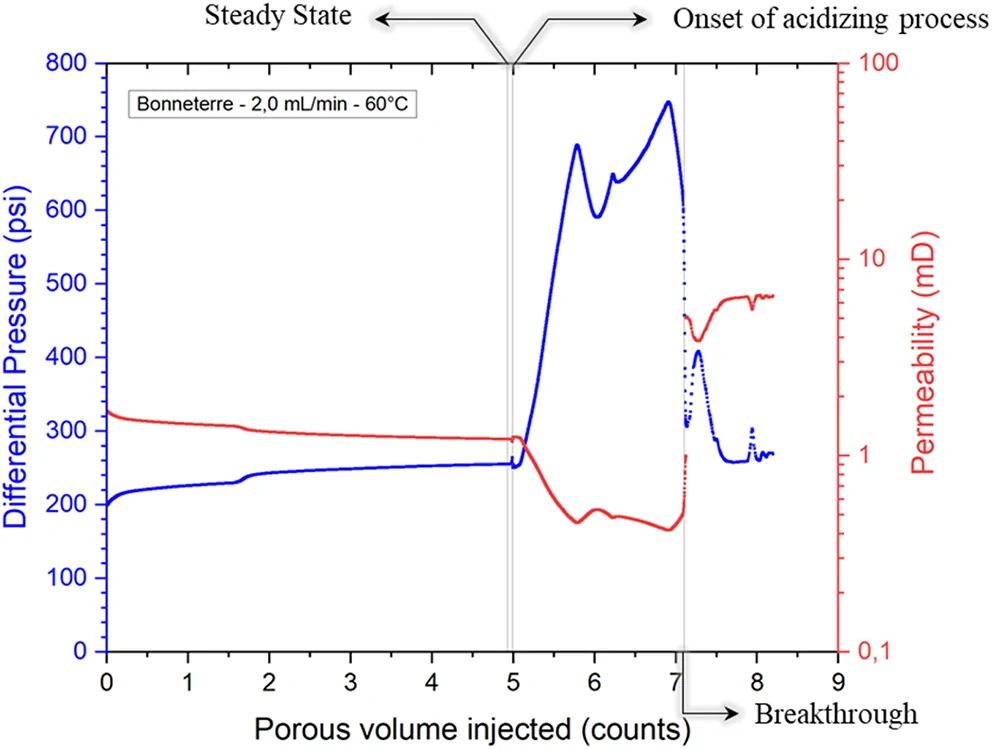
Differential pressure as a function of the amount of injected pore volumes relating to the acidizing of sample BTD-06 at 60 °C and a flow rate of 2 mL/min.

Figure 6a,b show an appreciable amount of solid residue, which suggests the formation of acid-insoluble reaction products, likely associated with silicate and aluminosilicate phases remaining after carbonate dissolution. The emergence of this residue may explain the limited permeability gain observed in certain dolomite samples after acidizing, in contrast to the significant permeability enhancements typically seen in limestone samples.^5,23^

**Figure 6:**
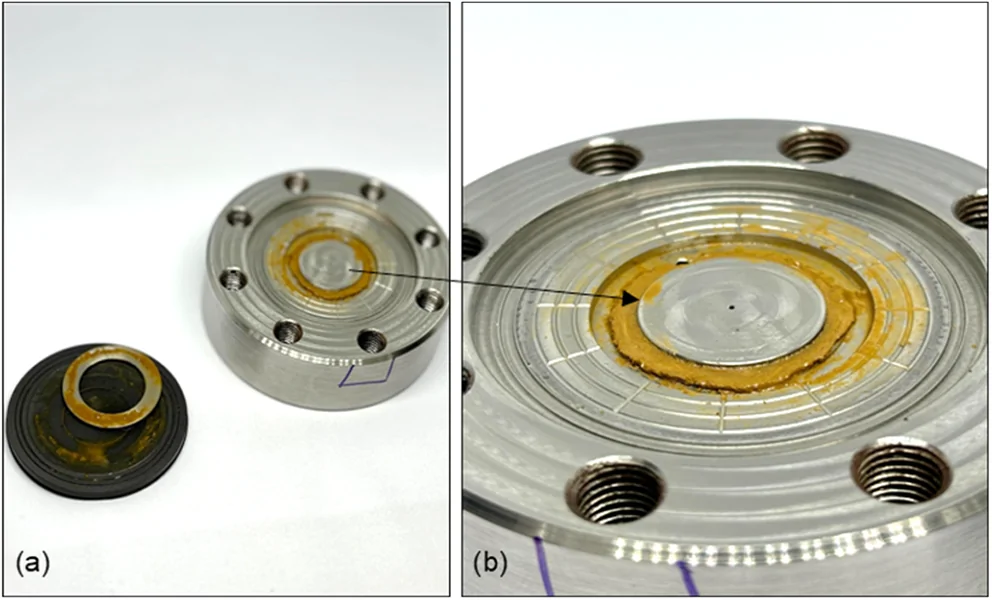
Backpressure valve after the acidizing experiments: (a) disassembled valve; (b) detail of the internal region showing the deposited material indicated by the arrow.

The solid reaction product generated by the interaction between Bonneterre Dolomite and the stimulation fluid was characterized by XRF, as described in Section 2.4. The analysis showed substantial depletion of CaO and MgO after acid exposure, consistent with the preferential dissolution of the dolomitic matrix reported in previous studies.^20,24,40^ In contrast, the remaining solid fraction was enriched in SiO_2_, Al_2_O_3_, and Fe_2_O_3_, confirming the persistence of acid-insoluble mineral phases after carbonate dissolution. The solid material visually observed in the backpressure system during the core flooding experiments is consistent with the presence of such insoluble phases. These residues may partially restrict pore throats or conductive pathways, thereby contributing to the limited permeability enhancement observed in some samples. However, because the amount of residue and the degree of obstruction were not quantified individually for each core flooding experiment, their specific contributions to the differences in apparent postacidizing permeability cannot be isolated from other effects, such as wormhole connectivity, intrinsic rock heterogeneity, and postbreakthrough pressure sensitivity.

#### 3.4.2 Analysis of the Rock Inlet and Outlet Faces after Acidizing

Figure 7 presents the inlet and outlet faces of BTD samples after being subjected to the flow test at 60 °C with different fluid flow rates. The image analysis allows the identification of significant changes on the plug surface as the acid flow rate increases, indicating differences in dissolution at the inlet surface of each sample.

**Figure 7:**
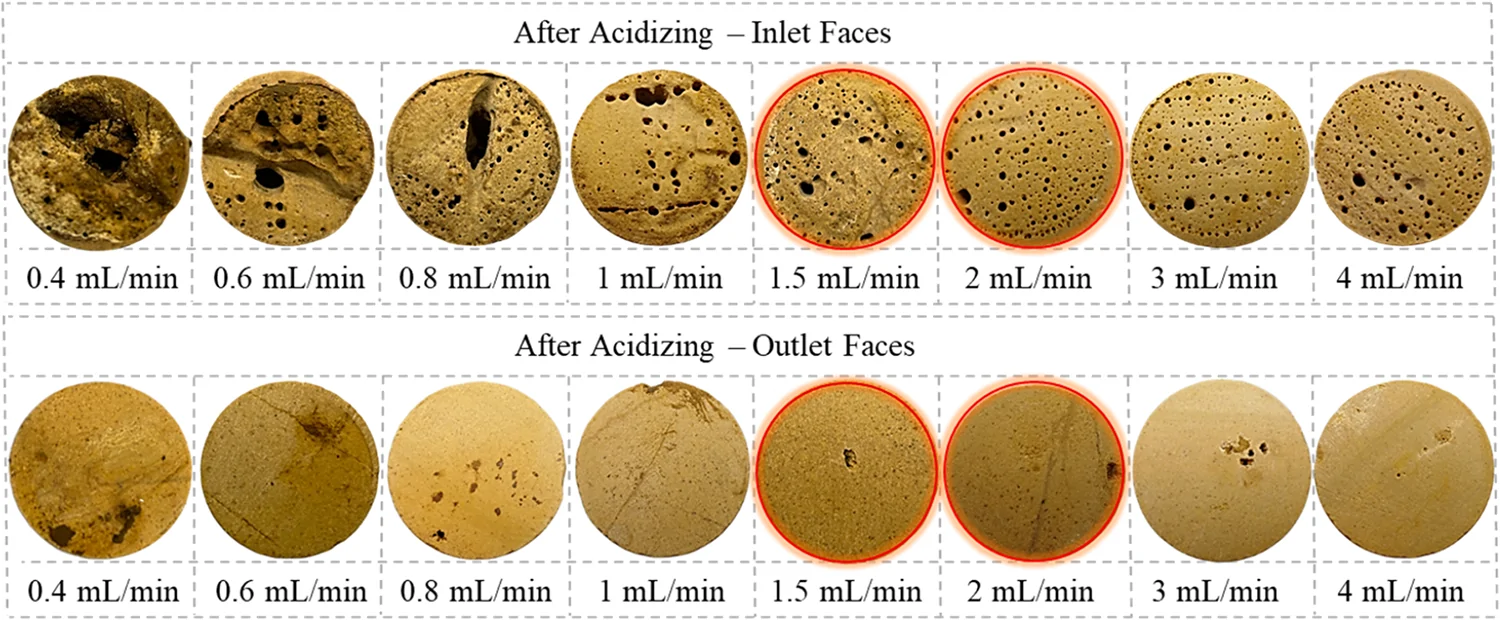
Inlet and outlet faces of the Bonneterre Dolomite samples after acidizing and their respective flow rates.

Acid-rock reactions can be described by three steps: mass transfer of H^+^ ions from the acid solution to the rock surface, surface reaction kinetics, and mass transfer of the reaction products from the rock surface to the bulk solution.^20^ The rate-limiting step defines the dissolution regime.

At lower flow rates (0.4 to 0.8 mL/min), the samples predominantly exhibited face dissolution and conical or poorly defined dissolution channels, indicating inefficient development of a single dominant wormhole. At intermediate flow rates (1.0 and 1.5 mL/min), greater preservation of the inlet face and the formation of intermediate-diameter channels were observed, a behavior consistent with that reported by Martyushev et al.^5^ The samples highlighted in red required lower acid volumes to reach breakthrough, which is consistent with more efficient wormhole development.

The formation of multiple pits on the inlet surface, observed at both low and high flow rates, suggests a greater difficulty in initiating dominant wormhole propagation compared to limestone, due to kinetic and mineralogical constraints.^23,40^ This behavior can be attributed to the heterogeneity and mineralogical composition of the BTD. Dolomite exhibits slower reaction kinetics compared to calcite, requiring a higher acid flux to achieve breakthrough, which directly influences the optimal PV_bt_ value.^20,23^ In this context, μCT images provide more conclusive information regarding wormhole formation.

#### 3.4.3 Computed Microtomography Analysis

μCT images of the carbonate rocks after acidizing allow for understanding the structure of the formed wormholes. Figure 8 shows the μCT images of the most representative views of the wormholes formed in BTD samples subjected to acidizing at 60 °C, using 15 wt % HCl with additives, across all tested injection flow rates. The images are presented in ascending order of flow rate, and conditions associated with higher efficiency, based on the resulting wormhole pattern, are highlighted in red.

**Figure 8:**
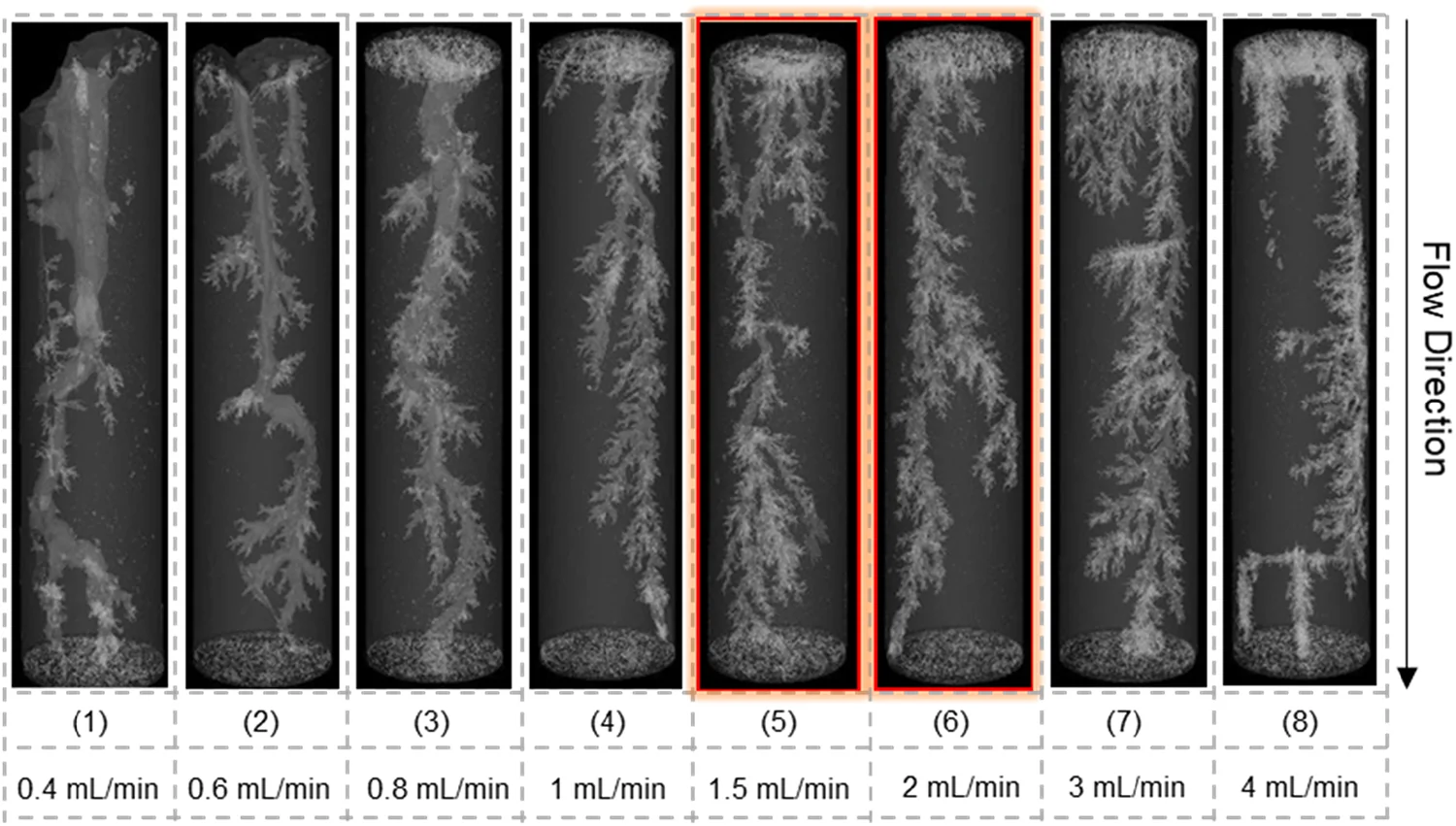
μCT images of the acidized Bonneterre Dolomite rock samples at 60 °C and their respective injection flow rates.

Overall, image analysis reveals that low acid injection rates promote dominant face dissolution, resulting in wormhole structures with larger volumes and geometries closer to a conical wormhole pattern. This behavior is clearly observed in images (1), (2), and (3) of Figure 8, where wormholes show larger diameters and a low quantity of developed branches, suggesting a more localized and less ramified dissolution process.

For BTD-01, the apparent extension of the dissolution structure toward the outlet in the post-test μCT image may also reflect displacement of residual acid during the initial brine postflush after the acid-injection stage had been terminated for safety reasons; therefore, this morphological observation should not be interpreted as experimentally confirmed breakthrough during acid injection.

As the flow rate increases, a significant change in the dissolution pattern is observed. Images (7) and (8), from Figure 8, show the formation of thinner and highly branched wormholes, with a marked presence of secondary channels on the inlet face. This highly ramified wormhole pattern is characteristic of higher injection rates, under which reactive transport is dominated by convection, promoting acid distribution over a larger area and resulting in a more complex channel network.

Images (5) and (6), at flow rates of 1.5 mL/min and 2.0 mL/min, respectively, show more uniform dissolution of the sample face compared to lower flow rates, along with the formation of dominant and well-developed wormholes under optimal and near-optimal conditions (2.0 and 1.5 mL/min, respectively). These results corroborate the PV_bt_ data associated with higher acidizing efficiency, indicating that these flow rates promote more efficient channel development than lower and higher injection rates.

The transition between dominant and highly branched wormhole morphologies may also have important geomechanical implications. A dominant wormhole concentrates dissolution within a relatively localized flow pathway, whereas highly branched networks distribute dissolution over a larger rock volume and introduce a greater number of internal discontinuities. Consequently, highly branched structures may promote more distributed weakening of the rock framework and greater local stress concentrations, potentially resulting in larger reductions in stiffness and strength than those associated with a single dominant channel.^13^ In heterogeneous dolomite, the presence of acid-insoluble mineral phases may further enhance spatial variations in the postacidizing mechanical response by creating local contrasts between dissolved and residual regions. Under reservoir depletion, such heterogeneity may become relevant as increasing effective stress acts on the acidized pore structure, potentially increasing susceptibility to localized deformation or pore-scale instability. However, because mechanical properties were not measured in the present study, these geomechanical implications should be treated as qualitative interpretations based on observed μCT morphologies and prior literature, rather than direct measurements of postacidizing mechanical behavior.

The morphology of the dissolution structures observed in the present μCT images can also be compared with previously reported behavior for limestone. Although the general sequence of dissolution regimes, including face dissolution, dominant wormhole formation, and highly branched structures, is observed in both carbonate lithologies as injection conditions change, important morphological differences have been reported. μCT studies by Martyushev et al.^5^ and Mustafa et al.^23^ showed that wormholes developed in dolomite tend to be larger and thicker than those formed in limestone. Similarly, Dong et al.^24^ reported that, although the overall wormhole geometry in dolomite is comparable to that observed in limestone, dolomite generally develops a larger dissolution-channel volume. These differences are consistent with the slower reaction kinetics of dolomite with HCl, which reduces acid utilization efficiency and requires a larger acid volume for channel propagation and breakthrough. In the present study, the relatively large face-dissolution regions and thick dissolution channels observed in Figure 8 are consistent with this characteristic dolomitic behavior. In contrast, limestone systems reported in the literature generally achieve effective channel propagation with lower acid consumption. Therefore, the μCT observations reinforce the conclusion that mineralogy affects not only the minimum PVbt and the optimum injection conditions but also the volume and geometry of the wormhole network developed during acid stimulation.

## 4 Conclusions

This study evaluated the acidizing behavior of Bonneterre Dolomite using 15 wt % HCl containing a corrosion inhibitor and an emulsion preventer under representative core flooding conditions at 60 °C. The combined use of core flooding experiments, rock and fluid characterization, and X-ray microcomputed tomography enabled relating PV_bt_ behavior to wormhole morphology and mineralogical features. Based on the results, the following conclusions can be drawn:

1. An injection rate of 2.0 mL/min, corresponding to a PV_bt_ of 2.03, was identified as the optimum condition in terms of acid-utilization efficiency for the tested dolomitic rock and fluid system. Under these conditions, a lower acid volume was required to achieve breakthrough while promoting the formation of a dominant, deeper wormhole. However, this optimum should not be interpreted as the condition that necessarily maximizes postacidizing conductivity, since the persistence or redistribution of acid-insoluble mineral phases may partially restrict the hydraulic benefit of the generated flow pathways.
2. The postacidizing analysis of the core faces helped clarify the dissolution patterns associated with different injection rates. Lower flow rates promoted dominant face dissolution, whereas higher flow rates led to the formation of multiple pits and more distributed dissolution, indicating less favorable conditions for the initiation and propagation of a single dominant wormhole.
3. μCT analysis confirmed the strong relationship between injection rate and stimulation efficiency, in agreement with the PV_bt_ trend described by the Buijse and Glasbergen model. Three main dissolution regimes were identified: predominant face dissolution at low flow rates; dominant and well-developed wormholes under near-optimal conditions (1.5 and 2.0 mL/min); and thin, highly branched channels at high flow rates, consistent with classical carbonate acidizing behavior.
4. The predominance of dolomite (98.5%), together with the presence of noncarbonate impurities such as silicates, iron, and aluminum, increased the complexity of the acid-rock interaction, consistent with reports in the literature indicating a higher acid requirement than that commonly observed in limestone systems.
